# Association between Cognitive Status and Physical Activity: Study Profile on Baseline Survey of the My Mind Project

**DOI:** 10.3390/ijerph13060585

**Published:** 2016-06-14

**Authors:** Cristina Gagliardi, Roberta Papa, Demetrio Postacchini, Cinzia Giuli

**Affiliations:** 1 Centre of Socio-economic Gerontological Research, Italian National Research Centre on Ageing (INRCA), Ancona 60124, Italy; c.gagliardi@inrca.it (C.G.); r.papa@inrca.it (R.P.); 2 Unit of Geriatrics, Italian National Research Centre on Ageing, Fermo 63023, Italy; d.postacchini@inrca.it

**Keywords:** elderly, life style, physical activity, dementia

## Abstract

*Background*: The incidence of people with dementia is expected to increase significantly in the coming years, but it seems that there is a relationship between an active lifestyle and cognitive decline. The present study aimed to compare the characteristics and engagement in the physical activity (PA) of three groups of Italian elderly with different cognitive statuses at baseline phase. *Methods*: Data were examined using the results from the “My Mind Project” on 305 community-dwelling Italians. The sample was comprised of 93 subjects with Alzheimer’s disease (AD), 109 with mild cognitive impairment (MCI) and 103 healthy elderly (HE). *Results*: Classification of subjects on the basis of Physical Activity Scale for the Elderly (PASE) score showed that 47% of HE performed the highest level of physical activity while 40% of AD performed the lowest level. MCI subjects were distributed quite homogeneously across the levels (*p* < 0.001). Physical activity such as walking and light sports was carried out mainly and more frequently by HE as compared to the others (*p* < 0.05). As regards functional status, AD presented worse conditions in basic and instrumental activities of daily living than the other groups (*p* < 0.001). *Conclusions*: Our results evidenced that subjects with cognitive decline had the tendency to engage in PA less than HE. In particular, age and education negatively affected engagement in PA.

## 1. Introduction

The incidence of people with dementia is expected to increase significantly in the coming years. This phenomenon will have profound repercussions on the world’s health system, in particular on the increase in medical, economic and social costs, both for the elderly and for their families. Currently, cognitive decline and dementia represent a major social and health problem, generating a strong impact on the functional abilities and dependence status of the patients. The prevention of these diseases might lead to a reduction in hospitalization and the use of specific drugs [1]. Despite this, there is a lack of programs and strategies providing help to subjects with dementia and other cognitive deficits preventing the onset of the disease.

Literature has shown that there is a relationship between lifestyle and cognitive decline. Indeed, an active lifestyle maintained after retirement can help the mental performance of the elderly [2]. It is well known that insufficient social engagement is associated with a low quality of life as well as many negative health outcomes, such as functional decline and mortality [3]. Physical activity (PA), even at moderate levels, and participation in recreational activities may have a protective effect on mental function and the incidence of dementia [4,5]. 

Longitudinal studies seem to confirm the association between PA and a lower risk of dementia. A study involving elderly without cognitive disorders, followed for more than five years, highlighted that the number of PA was inversely associated with dementia risk which further fell almost by half for those who were engaged in more than one activity [6]. In conclusion, we can suppose that the risk of dementia can be reduced in the presence of high levels of activity, and that the protective effect of PA is greatly enhanced by the association with mental activities, both social and cognitive.

In this context, the “My Mind Project”, a randomized intervention project ongoing in Italy, aims to use an interventional approach that combines cognitive training, education and advice about healthy lifestyle strategies to maintain cognitive reserves, engagement in PA, leisure activities and socialization [7].

The aim of the present study is to compare the lifestyle characteristics of three groups of Italian elderly with different cognitive statuses at baseline phase in order to preliminarily identify their engagement in PA and the correlated variables.

The changes in lifestyle characteristics will be subject to longitudinal analysis after the intervention.

## 2. Materials and Methods

### 2.1. Methods

The study design was a prospective randomized intervention project for the assessment of the effects of a comprehensive training in the elderly, using a multidisciplinary approach with three phases of follow-ups. Preliminary analyses were conducted on 305 Italian community-dwelling elderly subjects with mild cognitive impairment (MCI group), with Alzheimer’s disease (AD group) and without cognitive decline (healthy elderly, HE) living in the Marche region, recruited in the study “My Mind Project: The effects of cognitive training for elderly” (grant n. 154/GR-2009-1584108) from the Evaluation of Alzheimer’s Unit at the INRCA Hospital in Fermo. Recruitment and inclusion and exclusion criteria are reported elsewhere [7].

### 2.2. Ethical Approval

All procedures performed in studies involving human participants were in accordance with the ethical standards of the institutional and/or national research committee and with the 1964 Declaration of Helsinki and its later amendments or comparable ethical standards. The study was approved by the Institutional Ethical Committee (code SC/12/301) of INRCA. Informed consent was obtained from all individual participants included in the study.

### 2.3. Instruments

#### 2.3.1. Background Characteristics and Physical Activity Assessment

A specific questionnaire was developed to obtain information on smoking habits, alcohol consumption, dietary habits. Participants were asked at baseline whether they had smoked in their lifetime and whether they currently smoked. Three groups were formed to include: (1) current smokers; (2) ex-smokers and (3) non-smokers. Subjects were asked whether they had ever drunk alcohol and were assessed for type of drink (beer, white wine, red wine, liqueurs and spirits) and frequency. Subjects were also asked if they used to participate in any leisure activity, and if yes, they were asked to specify which one and the related weekly frequency in hours. Activities were then classified as social or solitary [8]. For the purpose of this study, a new dummy variable indicating participation or not in social activities was created.

The validated Italian version of the Physical Activity Scale for the Elderly (PASE) [9] was used to assess the amount of physical activity. The PASE consists of questions on occupational, household, and leisure activities over a one-week period. For each activity, the frequency (number of times per week) and the duration (number of hours) were asked. The total score is calculated by multiplying the frequencies (minutes and days per week) to specific weights for each kind of activity. Quartiles were used to classify people in different levels of physical activity (cut-off points were 51, 85 and 118); the first one indicated no to low physical activity and the fourth included people performing intense physical activity [10].

#### 2.3.2. Cognitive and Psycho-Social Aspects

Overall cognitive status is assessed by means of the Mini Mental State Examination (MMSE) [11].

The Geriatric Depression Scale (GDS-30) [12] was used to measure participants’ mood status.

Social networks and informal social support were measured using the Lubben Social Network Scale (LSNS; range 0–60) [13]. The scale assesses the extent of social contact with family and friends; for the purpose of the study the total score was classified in four categories, from “excellent” to “poor” [14].

#### 2.3.3. Assessment of Functional and Clinical Status

The Activities of Daily Living (ADL; range 0–6) [15] and Instrumental Activities of Daily Living (IADL; range 0–8) [16] were used to assess participants’ functional status; higher scores represent better functional status.

Data about clinical conditions are collected. In particular, presence of cardiovascular, respiratory, musculoskeletal diseases and diabetes, as well as BMI (Body Mass Index) was considered for this study. An aggregate score of the number of diseases was calculated. 

### 2.4. Statistical Analysis

Data were analyzed with SPSS 16 software (SPSS Inc., Chicago, IL, USA). Differences among groups (HE, MCI, AD) were assessed by Chi-square test for categorical variables and *t*-test or ANOVA for continuous ones. Two-sided tests were used. A value of *p* < 0.05 was considered for statistical significance.

## 3. Results

### 3.1. Subject Characteristics

A total of 305 subjects participated in the baseline assessment. Table 1 shows the main characteristics of the subjects by group (HE, MCI, AD). The majority of the subjects were female. A significant difference of age was observed among groups (*p* < 0.001). Healthy subjects had a higher education with respect to the other groups (*p* < 0.001).

Sixty-two percent of the participants never smoked, while 72% used to drink alcohol, without differences among groups (*p* > 0.005).

As regards functional status, both ADL and IADL significantly differed among participants (*p* < 0.001), with AD subjects presenting worse conditions in both measures. Mood status, evaluated through the GDS-30, was significantly poor for AD and MCI with respect to the HE (*p* = 0.042).

Self-evaluation of social support was significantly higher in HE as compared to the others (*p* = 0.001); about 15% of AD subjects gave a poor evaluation and are at risk of social isolation. This aspect is linked to participation in social activities, for which the same situation applies (*p* < 0.001).

### 3.2. Physical Activity

Classification of subjects on the basis of the PASE score showed that 47% of HE subjects were in the fourth quartile (highest level of physical activity) while 40% of AD were in the first quartile (lowest level); MCI subjects were distributed quite homogeneously across the levels (*p* < 0.001). 

Table 2 shows the different components of PASE and the mean value of each one by group. A main contribution was provided by the household activities (*i.e.*, housework, home repair, yard work and gardening) for all groups. Physical activity such as walking and light sports was carried out mainly and more frequently by HE as compared to the others (*p* < 0.05).

## 4. Discussion

The aim of this paper was to identify different lifestyle characteristics in three groups of elderly with dementia, MCI and without cognitive impairment. 

As expected, our results showed that subjects with cognitive decline (MCI and AD groups) had a tendency to engage in physical activity and social activities less than healthy elderly. In particular, age and education were negatively associated with engagement in PA, as also shown in our previous study [17]. Correlation analysis showed a significant relation between PA level and age in the MCI group.

Also, clinical conditions, psycho-social aspects (such as mood depression and perception of social support), and functional and cognitive status are related to engagement in PA [18,19]. Limitations in attention, verbal skills and communication skills can further contribute to the reduction of social interactions [20].

Previous studies showed that opportunities for active engagement in physical activity, including social activities, may decline with age, particularly in the presence of physical function declines and disability [20]. In accordance with this sentence, our results showed that subjects with higher disability (AD group) had a significant correlation between PASE and functional status, both in basic rather than in instrumental activities of daily living. On the other hand, the MCI group showed this relationship only for instrumental activities. In this respect, it was highlighted that changes in memory and executive functioning can be associated with instrumental activities of daily living in older adults [21]. Our results showed that obesity and overweight do not have a significant influence on this relationship.

The next step of the “My Mind Project” study will be to verify the causal role of the correlates in a longitudinal analysis. 

Some authors concluded that a structured PA program reduced major mobility disabilities in older adults at risk for disability, suggesting a mobility benefit from such a program in vulnerable older adults [22]. Likewise, the protective effect of social and recreational activities on dementia risk [5,23,24] may benefit the elderly with dementia through an activity program on behavioral symptoms, resulting in less restless behavior, a reduced use of medications, and improved nutrition [25].

The relationship between physical activity and psychological status and cognition in older subjects has been widely studied, showing that this relation could be reciprocal. Many authors appointed that PA may be beneficial for cognition because higher levels of leisure time PA and healthy eating seem to be protective against dementia [26,27]. 

PA is also considered as an intervention which decreases the burden associated with depression and cognitive impairment later in life [17]. 

## 5. Conclusions

Despite the increasing number of studies, the relationship between cognitive decline and reduced physical activity remains currently unclear and research is needed to better understand the causal association between cognitive function and physical activity. Research should also be a priority to support health policies in order to identify and validate prevention programs. The prevention of diseases linked to cognitive decline could lead to a reduction of costs not only for patients and their families, but also for the health and social welfare system.

Our analysis showed that elderly with cognitive decline engaged in physical and social activity less than those without cognitive deficits, with potential negative consequences on psychological conditions and functional status. The three groups presented different characteristics in terms of lifestyle profile, thus highlighting a range of opportunities for prevention. The effect of the comprehensive training used in the “My Mind Project”, which included psycho-education and advice for a healthy lifestyle, will be longitudinally analyzed in future papers. 

## Figures and Tables

**Table 1:** Characteristics of the subjects by group.

Subjects’ Characteristics	Healthy Elderly (*n* = 103)	MCI (*n* = 109)	AD (*n* = 93)	Total (*n* = 305)	*p*-Value
	Mean ± SD %	Mean ± SD %	Mean ± SD %	Mean ± SD %	
Age (years)	72.6 ± 5.9	75.9 ± 6.0	77.6 ± 5.3	75.3 ± 6.1	<0.001
Gender					0.069
Male	22.3	36.7	32.3	30.5	
Female	77.7	63.3	67.7	69.5	
Education (years)	9.6 ± 4.5	6.0 ± 3.5	5.1 ± 3.4	6.9 ± 4.3	<0.001
Kind of household					0.037
Alone	27.2	15.6	12.9	18.7	
With the partner	40.8	57.8	45.2	48.2	
With partner and children	8.7	6.4	11.8	8.9	
With other persons	23.3	20.2	30.1	24.3	
Smoking status					0.64
Smokers	9.1	5.0	9.8	7.9	
Ex-smokers	28.3	33.7	27.2	29.8	
Never smoked	62.6	61.4	63.0	62.3	
Alcohol consumption (yes)	71.7	73.3	70.7	71.9	0.92
BMI	26.4	25.5	25.6	25.85	0.208
PASE (quartiles)					<0.001
1° (0–51)	11.2	24.8	40.2	25.1	
2° (52–85)	17.3	28.7	27.2	24.4	
3° (86–118)	24.5	26.7	25.0	25.4	
4° (>119)	46.9	19.8	7.6	25.1	
Cardiovascular diseases (yes)	65.0	66.7	79.6	70.1	0.054
Respiratory diseases (yes)	6.8	1.9	6.5	4.9	0.182
Musculoskeletal diseases (yes)	61.2	52.8	66.7	59.9	0.127
Diabetes	4.9	8.3	7.5	6.9	0.585
Number of diseases	3.5 ± 2.1	3.4 ± 1.6	4.5 ± 1.7	3.7 ± 1.9	<0.001
ADL	5.9 ± 0.3	5.8 ± 0.4	5.2 ± 1.1	5.7 ± 0.7	<0.001
IADL	7.9 ± 0.5	7.5 ± 0.8	3.4 ± 2.0	6.3 ± 2.4	<0.001
MMSE	27.9 ± 1.1	25.8 ± 2.1	20.1 ± 4.1	24.8 ± 4.2	<0.001
GDS	7.9 ± 5.2	10.0 ± 5.8	9.2 ± 6.4	9.1 ± 5.8	0.042
LSNS					0.001
Poor	11.3	4.9	15.2	10.3	
Fair	16.3	24.5	27.2	22.6	
Good	60.2	68.6	55.4	61.6	
Excellent	12.2	2.0	2.2	5.5	
Participation in social activities (yes)	58.6	32.7	19.6	37.3	<0.001

**Table 2:** PASE components by group.

PASE Component	Healthy Elderly	MCI	AD
Walking **^¶^**	9.89	6.92	4.97
Light sports **^#,¶^**	3.86	1.21	0.45
Moderate sports	0.8	0.1	0.25
Strenuous sports	0.07	0.18	0.54
Muscle strength/endurance	0.28	0.00	0.00
Light housework **^#,¶^**	22.19	18.32	16.85
Heavy housework ^¶,^*	16.84	13.12	8.15
Home repair **^#,¶^**	18.98	11.58	8.48
Lawn/yard work	18.37	15.68	14.48
Outdoor gardening	9.39	8.91	6.09
Caregiving duties **^#,¶^**	12.14	5.2	1.9
Work/volunteer activities **^¶^**	4.07	2.08	1.6
Total score ^#,¶,^*	116.9	83.3	63.7

ANOVA comparing means across groups. **^#^** *p* < 0.05 between Healthy Elderly and MCI (Bonferroni *post-hoc* test). **^¶^** *p* < 0.05 between Healthy Elderly and AD (Bonferroni *post-hoc* test). * *p* < 0.05 between MCI and AD (Bonferroni *post-hoc* test).
